# Tetany caused by chronic diarrhea in a child with celiac disease: A case report

**DOI:** 10.1186/1757-1626-1-176

**Published:** 2008-09-23

**Authors:** Jaime Gabriel Hurtado-Valenzuela, Norberto Sotelo-Cruz, Guillermo López-Cervantes, Ana María Calderón de la Barca

**Affiliations:** 1 Departamento de Medicina Interna, Hospital Infantil del Estado de Sonora. Av. Reforma No. 355 Norte, Colonia Ley 57, Hermosillo 83000, Sonora, México; 2 Departamento de Patología, Hospital Infantil del Estado de Sonora. Av. Reforma No. 355 Norte, Colonia Ley 57, Hermosillo 83000, Sonora, México; 3 Departamento de Nutrición y Metabolismo, Centro de Investigación en Alimentación y Desarrollo, A.C. P.O. Box 1735, Hermosillo 83000, Sonora, México

## Abstract

There is no awareness about celiac disease (CD) in Mexico. A 2.9 year old mestizo boy was admitted to a Mexican hospital with muscle cramps and fine tremors. He suffered chronic diarrhea, abdominal distention, hypotrophic limbs, stunting and wasting, and presented hypocalcemia, anemia and high titers of serological markers. Diagnosis of CD was confirmed by a duodenal biopsy. After replacement of calcium and a gluten-free diet, the symptoms resolved within 6 weeks. After 2-months, serum analyses, anthropometric data as well as antibodies titers were normal after 4 years. CD screening tests are needed in chronic diarrhea for any ethnicity patients.

## Background

Celiac disease (CD) is an immune-mediated enteropathy triggered by ingestion of wheat gluten [1]. Although CD was considered to be a rare disease, nowadays it is recognized to involve all the ethnic groups with a worldwide prevalence of 1–2% [1-3]. However, CD prevalence is unknown in Mexico that has groups with complex genetics [4], and where there are no screening tests for CD in most hospital.

Celiac crisis consistent in acute diarrhea that leads to low serum ion concentrations and acidosis was common in children under two years [5]. Nowadays, the crisis is very rare in countries where CD is well known. Unfortunately, if not treated, symptoms of cramps and tetany may occur, usually associated with low calcium or magnesium serum levels [3].

A child case of a Mexican mestizo who presented hypocalcemic tetany as the initial symptoms of CD is described. The case is an example of the clinical course of untreated CD in a non Caucasian child and emphasizes the need to consider the analyses of serological markers for CD, in all the cases suffering from acute diarrhea with no signs of infections.

## Case presentation

**Figure 1:**
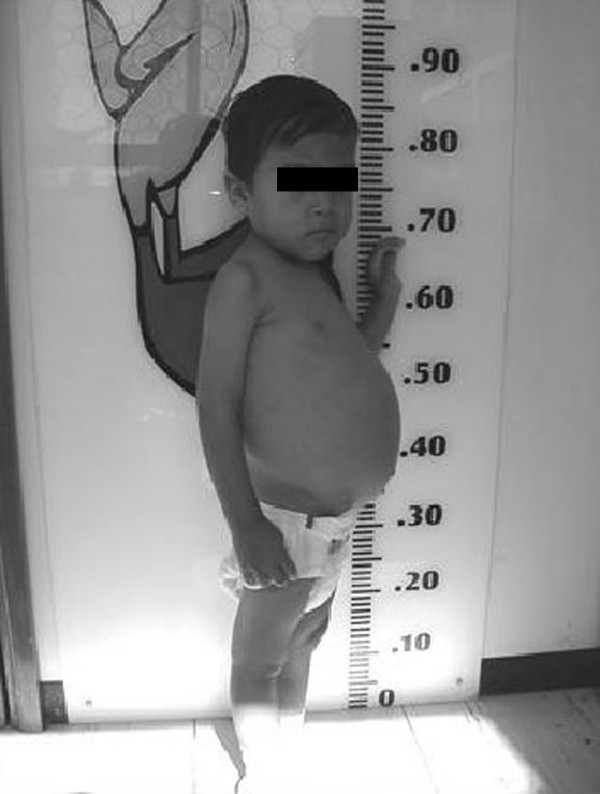
A 2 year and 11-months old boy with untreated celiac disease showing hypotrophy and abdominal distention.

A 2 year and 11-month-old mestizo boy was admitted to the Hospital Infantil del Estado de Sonora (Children Hospital of Sonora State, Mexico) with muscle cramps, fine tremors on hands and feet for the last 2 days. His mother reported chronic diarrhea for the last year with 6 hospitalizations because of diarrhea and dehydration. During physical examination, upper abdominal distention was observed (Figure 1), in addition to hypotrophic arms and legs and positive Chvostek's and Trousseau's signs. His weight and height were less than the fifth percentile for his age.

Blood count showed hemoglobin 101 g/L, white blood cells 8500/mmc, platelets 472,000/mmc, GVS 26 mm/h and reticulocytes 3%. Microcytic and hypochromic anemia was evident, with hematocrit 30%, mean globular volume 73 fL, serum iron 41 μg/dL and ferritin under 5 μg/L. Serum laboratory tests revealed hypocalcemia with total calcium of 5.7 mg/dL, sodium 133 mEq/L, potassium 3.2 mEq/L, chloride 103 mEq/L, glucose 82 mg/dL, AST 71 U/L, ALT 44 U/L, albumin 2.5 g/L, and C-reactive protein 0.5 mg/L. Billirubins, urine examination, and serial stool analyses for infectious etiologies, were negative.

**Figure 2:**
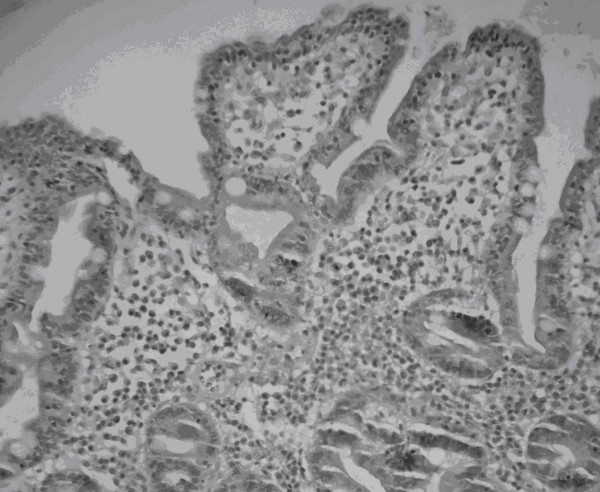
Photomicrography of the histology of a small intestinal biopsy specimen from a 2 year and 11-months old boy with untreated celiac disease.

The patient's tetany resolved after replacement of calcium and additional analyses were performed. IgA and IgG anti-gliadin antibodies as well as IgA anti-transglutaminase were positive, with index values of 3.5, 35.7 and 26.8, respectively. Index value was defined as the ratio of the absorbance of the test serum divided by the cut-off value [6]. Additionally, there was IgA reactivity to maize prolamins (zeins). The diagnosis of CD was confirmed by inflammatory cells infiltrate in the small intestinal mucosa and lymphocytes in the surface epithelium on duodenal biopsy, in a Marsh IIa/IIb classification (Figure 2). After initiation of a gluten-free diet and lactose-free milk (at the beginning), mineral and vitamins supplements, the patient's symptoms completely resolved within 6 weeks. The clinical and nutritional response to the gluten-free diet was excellent. In a 2-month follow-up period, he presented normal blood counts and serum biochemical analyses. Additionally, his weight and height were in the 75 percentile for his age, antibodies titers were normal and there were no further episodes of tetany or diarrhea over 4 years follow up period.

## Discussion

Tetany, a less common presenting feature of CD as seen in this case, was caused by severe hypocalcemia as a result of malabsorption [7]. The presence of recurrent diarrhea suggesting malabsorption was an important indication for making the correct diagnosis. IgA anti-gliadins antibodies were highly positive, showing that it is a sensitive test for CD in young children [1], as well as a very high index for anti-transglutaminase antibodies. Additionally, there was a mild IgA reactivity to maize prolamins, as it was previously detected [8]. Although the gold standard diagnostic for CD is a small-bowel biopsy with findings of villous atrophy, in this case, damage was mild (grade II of the Marsh classification). However, we found surface epithelium containing an increased population of intraepithelial lymphocytes, which is considered one of the morphologic hallmarks of CD [9].

In the Northwest of Mexico, where the present case is described, diet is mainly wheat-based; although maize is also used as in any Mexican population [10] and several of the weaning foods contain wheat and maize. Therefore, probably the onset of CD in this child occurred at weaning with introduction of cereals into the diet in a classical presentation. Awareness about maize-containing foods for complementary feeding could be done, due to the IgA reactivity to maize prolamins [8]. Because of the miss-diagnosis, the symptoms varied during the different periods, with diverse spectrum including cramps and tetany, which may occur, usually associated with low serum calcium or magnesium levels [3]. Definitive diagnosis of CD was clear in this case according to the European criteria [11]. The child's characteristics compatible with CD were: a) history and clinical presentation, b) serological tests, c) histological findings, d) obvious clinical and serological response to gluten-free diet, e) over two years old, and f) conditions as milk intolerance or gastroenteritis were excluded. Such CD pattern is common in developing countries, including short stature, diarrhea, abdominal distention, and anemia [11].

As illustrated in this case, CD should be considered in patients with tetany and diarrhea. Highly discriminatory markers for CD are needed to identify children with early CD onset. Appropriate test selection is important to obtain the most accurate diagnostic information. It is generally recommended that for elder children, IgA anti-endomisial tissue or anti-transglutaminase be used, but for very young children anti-gliadin antibodies may prove to be more sensitive [1]. Intestinal biopsy is required to confirm the diagnosis of CD.

## Conclusion

Awareness of CD should be increased amongst the healthcare sector for mestizo population, which is a complex mixture of genetics and constitutes the core of the Latin American populations. Although CD is apparently a less common illness in these populations, the new evidence indicates that it is not the case and deeper analyzes will be useful for better management, as well as reducing morbi-mortality related to CD in young children.

## Abbreviations

CD: Celiac disease.

## Consent

Written informed consent was obtained from the patient's parents for publication of this case report and accompanying images. A copy of the written consent is available for review by the Editor-in-Chief of this journal.

## Competing interests

The authors declare that they have no competing interests.

## Authors' contributions

JGHV conceived the study, and participated in its design and coordination and helped to draft the manuscript. NSC diagnosed the patient and drafted the initial manuscript. GLC performed the histological analysis and contributed to drafting the manuscript. AMC run serological tests for markers of CD and provided scientific input in the discussion of the article. All authors read and approved the final manuscript.
